# Friction Force Adjustment by an Innovative Covering System Applied with Superelastic NiTi Brackets and Wires—An In-Vitro Study

**DOI:** 10.3390/ma15124248

**Published:** 2022-06-15

**Authors:** Andrea Wichelhaus, Tena Eichenberg, Philip Gruber, Elias Panos Bamidis, Thomas Stocker

**Affiliations:** 1 Department of Orthodontics and Dentofacial Orthopedics, University Hospital, LMU Munich, Goethestrasse 70, 80336 Munich, Germany; gruberphips@icloud.com (P.G.); e.bamidis@posteo.de (E.P.B.); th.stocker@med.uni-muenchen.de (T.S.); 2 Orthodontic Specialist Practice Tena Eichenberg, Marienstraße 2, 89231 Neu-Ulm, Germany; eichenberg@kfo-ulm.de

**Keywords:** friction, covering, wire profile, elastic slot system, NiTi

## Abstract

The aim of this study was the investigation of polymeric coverings to adjust frictional forces between V-shaped wires and brackets, both made of superelastic NiTi. Adjustment of frictional forces is relevant for certain stages during orthodontic therapy. Coverings able to generate frictional forces when assembled to such brackets are additively manufactured. Six different internal widths of coverings were examined in three different environments: dry condition at room temperature (RT) or body temperature (BT), or artificial saliva (AS) at RT. The different coverings significantly affected the frictional forces for all media (*p* < 0.001). A correlation between internal width of the covering and resulting frictional forces was found. BT and dry environment showed the lowest friction forces for all samples. The highest force was found for two covering types at RT in AS, while the remaining four covering types showed the highest values in dry environment (*p* < 0.001). Friction could, therefore, be adjusted by variation of bracket covering clipped onto brackets, which is useful for orthodontic therapy. Coverings delivering higher friction provide dental anchorage, while coverings with lower friction can be used for tooth movement or purely esthetic reasons. It was shown that the variation of covering width may be used for adjustment of frictional forces.

## 1. Introduction

By moving a tooth with the aid of a fixed orthodontic appliance, part of the applied force is inevitably lost due to frictional forces. A distinction must be made between physical and biological factors, both affecting friction. Wire properties, such as material, surface condition and stiffness, as well as the design of the brackets, e.g., the width or depth of the slot, the bucco-lingual angle between slot and bracket-base (i.e., torque) and the amount of the applied force are important [1,2,3,4,5,6,7,8]. The type of ligation, which creates a force between wire and bracket, also has a major influence on frictional forces [9,10]. Biologically, saliva, plaque, food debris and corrosion also play a decisive role [11,12,13,14,15]. Friction in fixed appliances is, therefore, a multifactorial event. Taken together with the individuality of root extension, center of resistance, periodontal ligament, and bone structure, it is clinically challenging to predict tooth movement.

While high frictional forces have a negative influence on the efficiency of tooth movement along the archwire, a high frictional force may, on the other hand, serve anchorage purposes and thus exhibit beneficial effects on the therapy. Depending on the treatment stage in orthodontics, single teeth and tooth segments must be moved with the lowest possible friction in contrast to teeth that are used for anchorage [2].

In order to ensure efficient torque transmission for uprighting teeth and to meet esthetic requirements, a flexible superelastic NiTi bracket with a V-shaped slot geometry has been developed [16]. If the bracket wings are elastic, then micro-movements between archwire and bracket occur. This may contribute to a reduction in stresses due to infinitesimal deformations of the bracket wings during load application, e.g., during mastication, and leads to a decrease in frictional forces by reducing canting of the archwire in the slot or minimizing stick-slip effects [16].

Conventional bracket systems with rectangular slots must exhibit play between the archwire and the slot, which leads to an inaccurate transmission of the therapeutic torque to the tooth [17,18,19]. The manufacturers of orthodontic brackets do not explain how the width of the bracket slot is measured, while the allowable tolerances are defined by ISO Standard 13996, as −0/+0.04 mm [20]. In contrast, the initial torque transmission is play-free if a special V-shaped bracket and corresponding wire profiles are applied [16]. These attributes of the chosen bracket-wire system are important prerequisites for efficient vertical height leveling of teeth and successful subsequent tooth movement.

The aim of this study was to investigate whether the frictional force of the bracket-wire system can be adjusted independently by the clinician by means of a functional covering clip during different treatment stages or at localized parts of the orthodontic appliance. Additionally, the application of well-defined ligation forces generated by the covering allows for a much more accurate adjustment of frictional forces compared with manual ligation procedures. For this purpose, the frictional forces induced by different versions of the covering system were measured and their suitability was demonstrated.

## 2. Materials and Methods

**Figure 1:**
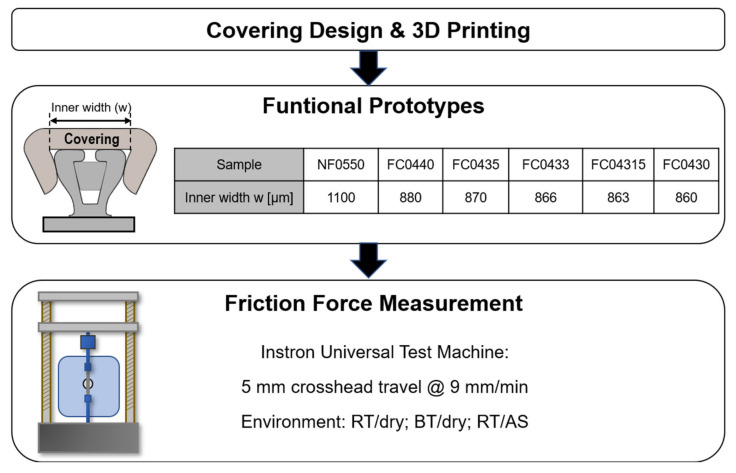
The applied workflow for the present in-vitro study consists of the design phase, followed by 3D printing of functional samples with the main parameter “inner width” shown in the table. The friction force measurements were carried out in a universal testing machine under the environmental conditions as shown above (RT: room temperature; BT: body temperature, AS: artificial saliva).

The workflow applied to this study is outlined in the following scheme (Figure 1). A detailed description is given in the following subsections.

### 2.1. Covering Design and 3D Printing

**Figure 2:**
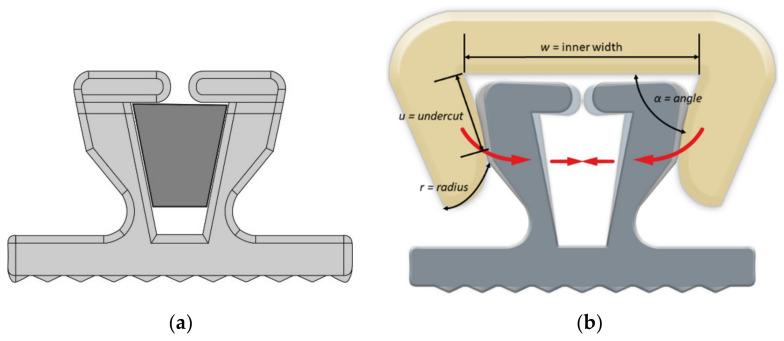
(**a**) In the superelastic NiTi bracket, the archwire is in contact with the wings, but does not touch the bottom. (**b**) Schematic drawing of the covering and its different design parameters. The covering compresses the wings to different degrees, depending on the inner width and design, allowing for better friction control.

The covering was designed using Autodesk Inventor 2015 (Autodesk, San Rafael, CA, USA) with the geometry based on a V-slot bracket (redsystem GmbH, Munich, Germany) (Figure 2a). Variable design parameters such as radii, angles, width and undercuts (Figure 2b) were implemented to change the appearance and performance of the covering. For the functional prototype, only the relevant parameter “width” was changed in the present study.

The functional prototypes were additively manufactured by means of a bottom-up Formlabs Form2 stereo lithography 3D printer (Formlabs, Somerville, MA, USA). The printing material was Formlabs Grey FLGPGR02 3D printing resin (Formlabs, Somerville, MA, USA) consisting of urethane dimethacrylate and methacrylate monomers. Vertical layer thickness (printer’s z-axis) was set to 25µm. After printing, the prototypes were washed with isopropyl alcohol (90%) for 10 min and post-polymerization was performed in a Kulzer Unilux AC, 200 W UV oven (Kulzer GmbH, Hanau, Germany) for 40 min. Production-related dimensional deviations originated mainly from the shrinkage due to 3D printing [21] and the covering’s geometry being close to the printer’s resolution.

### 2.2. Functional Prototypes

The dimensions of the covering types are summarized in Figure 1. The variation of the inner width (parameter “w”) was intended to induce varying amounts of frictional force based on the lateral pressure that the clip generated. Therefore, the widest of the covering types “NF0550” was designated as non-friction (=NF) covering. The different friction-inducing coverings (=FC) were designated as FC0440, FC0435, FC0433, FC04315 and FC0430. The digits following FC or NF, respectively, represent half of the inner width of the covering (w/2) (Figure 2b).

### 2.3. Friction Measurement

**Figure 3:**
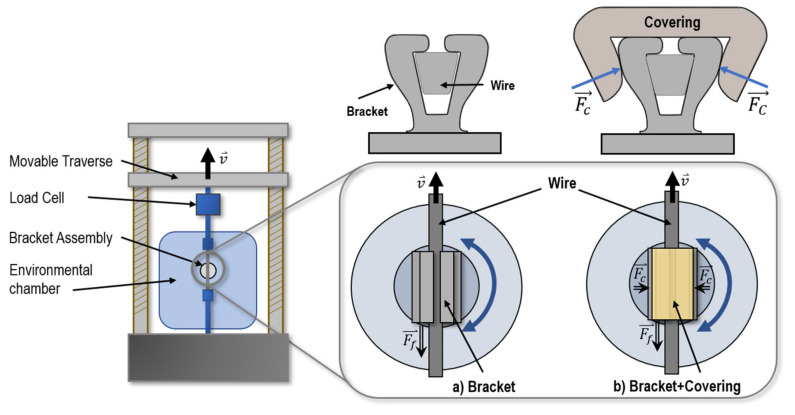
Specimen assembly for friction measurement. The superelastic NiTi bracket was centered and glued onto a hexagonal screw affixed play-free to a ball-bearing axis and was used to minimize canting of the wire. The superelastic NiTi wire (active A_f_-temperature: 18 °C) was connected to the force sensor of the universal testing machine and moved 5 mm upwards during frictional force measurements. The picture indicated with the letter (**a**) shows the bracket without covering. In the picture on the right, (**b**), a friction-increasing covering was applied to the bracket. Furthermore, the direction of the various force vectors are shown in the figure: F_f_ is the measured friction force, while F_c_ is the force induced by the covering to the bracket-wire complex.

In order to prepare for the friction force tests, a V-shaped archwire made from superelastic NiTi (active A_f_-temperature: 18 °C) was placed into a V-slot bracket also made from another batch of superelastic NiTi alloy (active A_f_ temperature: 8 °C; Ni-content: 55.85 wt %). Subsequently, one of the printed covering samples was mounted on the bracket-wire assembly. Three bracket prototypes and three V-shaped archwire prototypes were used for the measurements. The bracket was mounted onto a low friction ball bearing axis machined from stainless steel (Figure 3), which assured parallel alignment of wire and bracket slot [22]. The wire was mechanically connected to an Instron 2530 series 100 N static load cell within an Instron 4444 universal testing machine (Instron, Norwood, MA, USA).

After alignment of bracket and wire with the machine axis, the wire was pulled through the bracket-wire covering assembly by means of the Instron’s traverse movement while the opposing force was measured (Figure 3). This opposing force was interpreted as frictional force or resistance to sliding. Bluehill2 materials testing software (Instron, Norwood, MA, USA) was used for data logging and machine control. For the test procedure the following parameters were applied: 5 mm crosshead travel, and speed 9 mm/min, which were in accordance with parameter sets found in literature [23,24]. The test conditions were defined to be fully in-vitro. Therefore, the combination of test parameters was chosen to reflect, at least to a certain extent, the variety of media in the oral environment. The environmental conditions during the tests were defined as: (1) body temperature (BT) and dry; (2) room temperature (RT) and artificial saliva (AS); (3) RT and dry. These conditions were chosen to isolate the effect of the different environmental test parameters from each other. BT tests were performed at (36 ± 1) °C in a temperature chamber equipped with a REX-C100 (RKC Instrument Inc., Tokyo, Japan) temperature controller.

### 2.4. Statistical Analysis

All permutations of bracket and archwires were measured and the resulting values were averaged to reduce prototype state related dimensional deviations. The descriptive statistics included median, minimum and maximum values for each testing group. The dataset was not normally distributed. Hence, Kruskal-Wallis tests and Bonferroni post-hoc tests were used to compare and indicate significant differences between the effect of different coverings on the frictional force values. Calculations were carried out using IBM SPSS 27 (IBM, Armonk, NY, USA), with a significance level of α = 5%.

## 3. Results

The different coverings significantly affected the frictional forces measured for all media tested (*p* < 0.001, Table 1).

Except for two subgroups (FC04315 at 36 °C and FC0433 in artificial saliva at room temperature), there was a correlation between reducing the internal width of the covering (NF0550-FC0430) and the resulting frictional forces. A reduced covering width resulted in higher forces due to an increase in friction between archwire and bracket.

**Figure 4:**
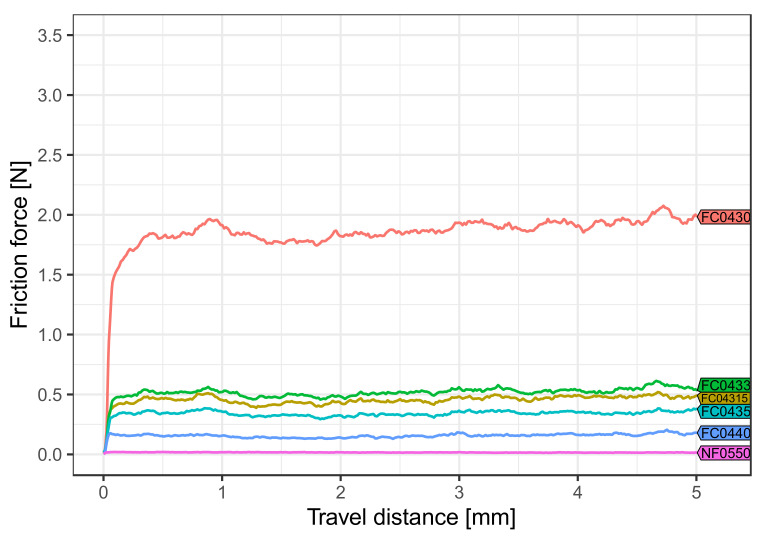
Effect of body temperature (36 ± 1 °C) and dry storage. The average intensity of friction force was reduced for the V-shaped archwire and V-slot in combination with the different coverings. Coverings able to generate frictional forces showed higher values (2.0 N) than those without this ability (0.1 N).

For each covering type tested, statistically significant differences between all media were found (*p* < 0.001). Body temperature and dry environment showed the lowest friction values for all coverings tested (Figure 4). 

**Figure 5:**
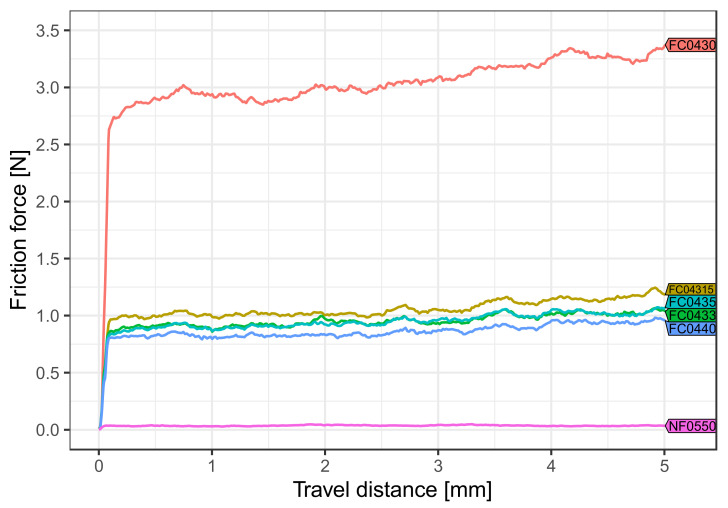
Effect of artificial saliva at room temperature. Whereas a low amount of frictional force occurred with covering NF0550, different friction force values were shown depending on the design. Only coverings FC0440 and FC0435 had higher friction force values in artificial saliva than in dry conditions.

**Figure 6:**
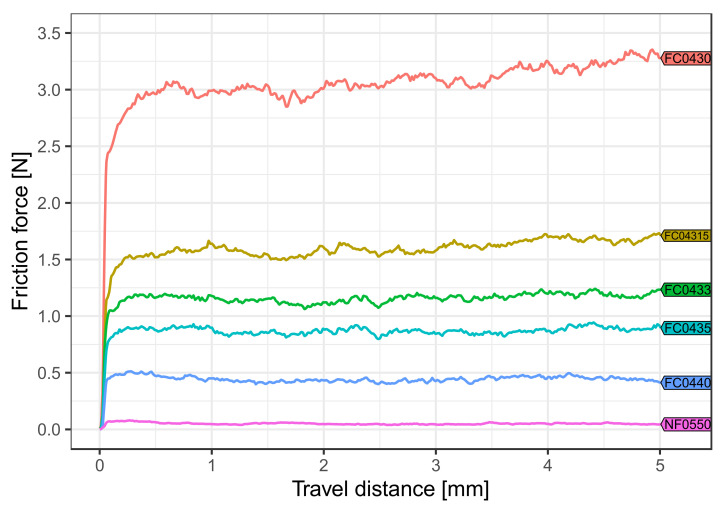
Effect of dry storage at room temperature. Depending on the design and inner width of an additional covering, average forces of 0.4–3.1 N were generated. The V-shaped archwire with V-slot showed a frictional force close to zero with non-friction covering NF0550 applied.

At room temperature, FC0440 and FC0435 showed the highest values in artificial saliva (Figure 5), while NF0550, FC0433, FC04315 and FC0430 delivered the highest values in dry environment (Figure 6).

For each medium tested, the pairwise comparison between the frictional force values of the different coverings (Table 1) revealed that almost all covering types were significantly different from each other (*p* < 0.001). Only coverings FC0433 and FC04315 at body temperature (Figure 4) and FC0433 and covering FC0435 (*p* = 1.0) in artificial saliva (Figure 5) showed insignificant statistical differences in their friction behavior. Overall, the lowest friction force value was obtained with NF0550 regardless of temperature and influence of saliva, whereas FC0430 exhibited the highest friction values.

## 4. Discussion

Friction in brackets is known to be a complex and system-depending variable, with the main factors being bracket design and wire dimensions [2,25,26,27,28]. High frictional forces hinder sagittal movement but allow for better anchorage, whereas tooth movement along the archwire is facilitated if low frictional forces are obtained [2,4,10]. According to the results presented in this study, frictional forces can be adjusted as needed during the phase of the orthodontic therapy by means of a corresponding polymeric covering clip. Thus, the bracket covering can be used as a real alternative to a conventional steel or elastics method of ligation depending on the clinical indication. This may be suitable for targeted dental anchorage. If superelastic NiTi brackets with V-shaped slot geometry are used, active and passive properties can be controlled by means of a functional covering, creating a defined amount of frictional force.

The amount of frictional force provided by the bracket and its covering depends on the wire dimensions [2,29]. Based on the given covering design, friction can only be applied in combination with a slot filling V-shaped archwire. It is obvious that the friction force generated by the coverings is higher if used with a slot-filling archwire rather than with a non slot-filling one, e.g., a round wire applied during leveling in the initial therapy stage. The combination of V-shaped slot geometry with the corresponding wire shape allows the play-free transmission of torque [16]. The addition of the presented covering adds the functionality of friction adjustment without affecting full torque transmission.

In an Angle Class I case with minor crowding (“lack of space for teeth”) and/or retruded teeth, as well as in cases with reciprocal gap closure, low friction is usually desired. In these cases, the therapy can be performed with a non-friction covering, serving esthetic purposes only. In patients with Angle Class II, III (anteroposterior discrepancy in the first molar position) and patients with crowding, that needs to be resolved in sagittal direction, dental anchorage is an important aspect. The present study showed that friction can be controlled just by changing the width of the covering.

Elastic deformation of the archwire causing resistance to sliding (binding) and plastic deformation of the archwire stopping the sliding (notching) usually occur under clinical conditions and can resist bodily tooth movement [28]. They are at least partially compensated by two factors: one is intrinsic to the applied system due to the superelastic property of both wire and bracket being made of NiTi (active A_f_ temperatures below room temperature), while the other is due to the experimental setup. Generally, the measured force resisting sliding *F_RS_* of the archwire moving within the bracket-slot is composed of the classical sliding friction *F_FR_*, which is generated by the normal force, binding *F_BI_* and notching *F_NO_* [28]. The parameters *F_BI_* and *F_NO_* increase their influence on *F_RS_* when the contact angle between bracket slot and archwire is increased:

(1) $$F_{RS}=F_{FR}+F_{BI}+F_{NO}$$

However, binding and notching are negligible for a passive parallel alignment between bracket slot and archwire, as being the case in this study. Herein, occurrence of binding and notching was eliminated by the alignment of the bracket to the archwire in the applied friction test. Thus, only *F_RS_* was relevant in this experimental setup. Clinically, binding and notching are also reduced by the elasticity of the superelastic NiTi and the related deformability of the bracket’s wings. 

The V-shaped archwire was pulled vertically through the bracket and neither torque was transmitted nor were other force vectors applied. The values of the frictional forces of the covering NF0550 in our study were comparable to those of passive self-ligating brackets [2]. It was shown that at 36 °C body temperature, the friction values were reduced by more than 50%. This could have been induced by the temperature-dependence of the superelasticity of the NiTi-brackets, as well as the V-shaped archwire. When the ambient temperature was increased to 36 °C, the NiTi bracket became more rigid and the force required to compress the bracket wings increased, leading to a possible decrease in measured frictional forces [30,31,32]. In this case, an increase of 7 MPa/K temperature change or 20–25% was to be expected according to the Clausius-Clapeyron relation [33]:

(2) $$\frac{d\sigma }{dT}=\frac{\Delta H}{T_{0}}\cdot \Delta \epsilon$$

This equation is used to describe the system’s behavior during a phase transformation. *dσ*/*dT* describes the stress-versus-temperature gradient, ∆*H* the transformation-enthalpy, *T*_0_ the equilibrium temperature of the martensitic transformation, and ∆*ε* the change in strain.

The thermal expansion of the polymeric coverings at 36 °C was considered to be marginal, since it would also reduce the friction caused by the coverings. Therefore, it can be assumed that the reduction in the measured frictional force values at 36 °C may mainly result from the increase in stiffness of the superelastic NiTi bracket rather than the thermal expansion. Over time, the thermally induced creep in the polymeric covering may also reduce its compression of the bracket and, therefore, affect friction, which so far has not been the subject of the research. 

As part of the surrounding medium, saliva can either act as lubricant [27] or adhesive [34], depending on the level of immersion of the bracket in saliva. Apart from two exceptions showing higher friction values, the measured wet friction values were lower than in the dry environment. Overall, the influence of artificial saliva was high, depending on the covering width. The relatively similar values of the frictional force for all other coverings could indicate that a fluid film of saliva was retained between the covering and bracket, acting as a lubricant. Smaller width coverings, such as FC430, could squeeze the fluid film out or even prevent the saliva from entering the gap between the covering and bracket. Therefore, high frictional forces are to be expected. The widest coverings did not exert any additional force perpendicular to the bracket and archwire, respectively. In consequence, these coverings exhibited almost no frictional resistance. Further work will be necessary to understand the chemical stability of the coverings in the oral cavity. A suitable, saliva-resistant polymer approved for medical devices such as, for example, PEEK should be found to replace the in-vitro covering material tested here. The swelling behavior in saliva and biofilm accumulation properties should also be investigated together with temperature-related property changes. Further research is needed to simulate the condition in the oral cavity, because friction is influenced by many intraoral factors [9,10,11,12,13,14,15]. Nevertheless, the focus of this study was to evaluate the effect of a new combination of covering and bracket on friction in vitro.

The experimental setup was an in-vitro idealized technical system. Prior to application of the covering, the V-shaped archwire was fixed free of stress in the bracket slot and, therefore, can be considered passive [28]. Due to the bracket being mounted on a ball bearing axis, the alignment allowed the isolated view of the frictional forces exerted by the coverings, independent of any bracket rotation [2,22]. Rotation of the bracket would result in lateral canting of the archwire in the bracket slot and would affect the friction by causing binding and notching. In addition, due to the self-adjusting property of the experimental setup, the parallel alignment of the bracket slot and wire was independent of the operator’s skills.

Further research work by means of finite element analysis (FEA) will be conducted to simulate the stresses and stress distributions generated by the covering on both the bracket as well as the archwire. In addition, tribologically relevant information about surface morphologies, roughness and effects of changes of said parameters will need to be addressed. However, for the clinical application of the developed polymeric covering, traditional tribological questions concerning wear and tear are of minor interest due to the fact that the archwire is changed every couple of weeks and the total travel distance of the involved components in tribological contact is only in the range of 10 mm or less over the entire lifetime.

## 5. Conclusions

- With the different coverings tested in this study, it was possible to control friction force levels independently of the clinician;
- Coverings delivering higher frictional forces are useful for dental anchorage, while coverings providing lower frictional forces can be used for facilitating tooth movement;
- Given the material properties of superelastic NiTi, temperature had a significant effect on the resulting frictional forces;
- In an Angle Class I case with minor crowding and/or retruded teeth, as well as in cases with reciprocal gap closure, low friction is usually desired. In this case, it should be possible to perform the treatment with a friction-free covering serving only esthetic purposes;
- In patients with Angle Class II, III, and patients with crowding that needs to be resolved in sagittal direction, dental anchorage is an important aspect. Friction can be controlled by changing the width of the covering.

## Figures and Tables

**Table 1:** Median friction between the six types of coverings and wires in different environments.

Temperature/Medium	Friction [N]—Median [Min; Max]					
	NF0550	FC0440	FC0435	FC0433	FC04315	FC0430
RT/dry	0.02 [0.01;0.42] ^Ac^	0.38 [0.10;1.08] ^Bb^	0.96 [0.31;1.44] ^Cb^	1.11 [0.55;1.80] ^Dc^	1.59 [0.96;2.39] ^Ec^	3.09 [1.79;4.01] ^Fc^
BT/dry	0.01 [0.00;0.06] ^Aa^	0.12 [0.01;0.64] ^Ba^	0.32 [0.05;0.77] ^Ca^	0.52 [0.02;1.27] ^Da^	0.40 [0.11;1.21] ^Da^	1.96 [0.39;2.96] ^Ea^
RT/AS	0.01 [0.00;0.14] ^Ab^	0.86 [0.23;1.60] ^Bc^	0.96 [0.34;1.46] ^Cc^	0.97 [0.31;1.62] ^Cb^	1.01 [0.32;2.29] ^Db^	2.87 [2.15;4.41] ^Eb^

Differing superscript letters indicate statistically different median values. Capital letters (A–F) describe differences between the differently-sized coverings. Lowercase (a–c) letters indicate differences within one type of covering comparing the different environments.

## Data Availability

The original data set can be provided upon request.
